# Self-Reported Olfactory Dysfunction and Diet Quality: Findings from the 2011–2014 National Health and Nutrition Examination Survey (NHANES)

**DOI:** 10.3390/nu13124561

**Published:** 2021-12-20

**Authors:** Shristi Rawal, Valerie B. Duffy, Lauren Berube, John E. Hayes, Ashima K. Kant, Chuan-Ming Li, Barry I. Graubard, Howard J. Hoffman

**Affiliations:** 1 Department of Clinical and Preventive Nutrition Sciences, Rutgers School of Health Professions, 65 Bergen Str., Newark, NJ 07107-1709, USA; 2 Department of Allied Health Sciences, University of Connecticut, 358 Mansfield Rd., Storrs, CT 06269, USA; valerie.duffy@uconn.edu; 3 Department of Population Health, New York University Grossman School of Medicine, New York, NY 10016, USA; lauren.thomas@nyulangone.org; 4 Sensory Evaluation Center, The Pennsylvania State University, 220 Erickson Food Science Building, University Park, PA 16802, USA; jeh40@psu.edu; 5 Department of Food Science, College of Agricultural Sciences, The Pennsylvania State University, 220 Erickson Food Science Building, University Park, PA 16802, USA; 6 Department of Family, Nutrition, and Exercise Sciences, Queens College, City University of New York, Flushing, NY 11367, USA; ashima.kant@qc.cuny.edu; 7 Epidemiology and Statistics Program, Division of Scientific Programs, National Institute on Deafness and Other Communication Disorders, National Institutes of Health (NIH), 10 Center Dr., Bethesda, MD 20892, USA; chuan-ming.li@nih.gov (C.-M.L.); hoffmanh@nidcd.nih.gov (H.J.H.); 8 Division of Cancer Epidemiology and Genetics, Biostatistics Branch, National Cancer Institute, National Institutes of Health, Bethesda, MD 20894, USA; graubarb@exchange.nih.gov

**Keywords:** olfaction, diet quality, energy density, dietary diversity, anosmia, hyposmia, epidemiology, added sugar, saturated fat, obesity, NHANES

## Abstract

We identified associations between self-reported olfactory dysfunction (OD) and dietary attributes in participants aged ≥40 years (*n* = 6,356) from the nationally representative 2011–2014 National Health and Nutrition Examination Survey (NHANES). The chemosensory questionnaire and 24-h dietary recalls were administered by trained interviewers. OD was defined as self-report of either smell problems in the last year, worse smell relative to age 25, or perceiving phantom odors. Dietary outcomes included Healthy Eating Index 2015 score (HEI) with adequacy and moderation components (higher scores indicated higher diet quality), dietary diversity, energy density, and intake of major food groups. Survey-weighted linear regression models estimated OD–diet associations, adjusting for socio-demographic, lifestyle, and clinical factors. Adjusted mean difference (95% CI) between those with versus without OD, showed that adults with OD had significantly lower HEI moderation score (−0.67 (−1.22, −0.11)) and diets higher in energy density (0.06 (0.00, 0.11)), and percent energy from saturated fat (0.47 (0.12, 0.81)), total fat (0.96 (0.22, 1.70)), and added sugar (1.00 (0.33, 1.66)). Age and sex-stratified analyses showed that younger females (40–64 years) primarily accounted for the associations with diet quality and total/saturated fat intake. These findings inform dietary screening and recommendations for adults who report OD, including those experiencing transient or persistent smell loss with COVID-19.

## 1. Introduction

The coronavirus-19 (COVID-19) pandemic has brought important attention to the chemical senses and the role that poor diet and related conditions, such as obesity, play in the risk of COVID-19 infection. Loss of sense of smell, taste and/or possibly chemesthesis is a hallmark sign of COVID-19 [1,2,3,4,5], emerging early in the disease’s course and affecting most infected individuals. Self-reported olfactory dysfunction (OD) is an important indicator of a smell problem and is one of the best predictors of a positive COVID-19 status [5,6,7]. The sense of smell plays a central role in the composite perceptual experience of food flavor, and accumulating evidence indicates its strong potential to influence diet selection, diet quality, and energy intake [8]. An important source of data on self-reported OD and diet comes from the chemosensory component first included in the U.S. National Health and Nutrition Examination Survey (NHANES) in 2011. This manuscript presents a detailed assessment of associations between self-reported OD and measures of diet quality and energy intake in this nationally representative sample, while controlling for multiple demographic, health and lifestyle behaviors.

Clinicians and public health professionals widely accept self-report as an inexpensive and efficient means of assessing individual and population characteristics that are strong and independent predictors of health outcomes (see [9,10]). Importantly, self-report assesses the individual’s perception of illness, which may be different from a clinician’s assessment of disease [11]. Broadly, self-reported health status, usually from a single question with responses ranging from excellent to poor, is a valid and reliable measure that correlates reasonably well with clinical assessments of measured health [12,13]. Similarly, self-reported smell function is strongly correlated with measured olfaction, and has previously been shown to be associated with important health outcomes such as increased risk of 10-year mortality [14] and dementia [15]. In 6,000+ patients from an otolaryngology practice in Dresden, Germany, self-rated olfaction showed good correlation with measured function [16] with up to 80% accuracy when compared against an odor identification test [17]. Self-rated olfaction also shows good correlation with measured function in patients undergoing chemotherapy across multiple studies [16]. In addition, a large Swedish population-based prospective study (*n* = 903) conducted among older adults without cognitive impairment found that subjective olfactory decline was significantly associated with decline in odor identification function over a 10-year time period [18].

Importantly, self-rated health provides an individual’s perspective on how they view their health within the context of their environment. General assessments of health are related to individual factors such as personality [19] as well as population differences and health disparities [20]. Misperception of poor health has been a focus in health education, with the idea that if individuals have a more accurate perception, then they will act upon this information differently and for the better [21]. Some individuals are indeed unaware of smell impairment—one study of over 9,000 individuals (ages 5 to 90s) found up to 3.4% with poor measured olfaction (anosmia, severe hyposmia) even though they reported a normal sense of smell [22]. Those unaware ranged from children to older adults and included males and females, and did not voice being bothered by the missing the sense of smell from a health or functional perspective. However, it is also worth noting odor identification tasks also may overestimate the level of OD [17]. People may misidentify a specific odor (due to unfamiliarity or memory issues), yet may still be able to smell something and feel they do not have an olfactory problem. In a large, representative cohort study of older adults in the U.S., individuals who lacked awareness of their OD were more likely to be older, Black, never married, and to have poorer cognitive function than those who recognized the dysfunction [23]. These findings suggest the importance of examining self-reported OD and diet associations while controlling for demographic, health, and lifestyle variables.

While multiple studies suggest that smell alterations are associated with differences in dietary selection, the empirical evidence remains highly inconsistent, and little is known about the specific dietary patterns associated with OD. In case-control [24,25] and descriptive [26] studies, otherwise healthy U.S. adults (ages 24 to >60 years old) who sought treatment for olfactory disorders at National Institutes of Health-funded clinical research centers reported less enjoyment from eating, but did not report insufficient intake of vitamins/minerals; rather, women were more likely to report weight gain. Notably, there were also substantial sex differences in diet [26]—across the entire age range, women with olfactory loss were more likely to report changing their dietary behaviors, employing food-related strategies, eating more in response to the loss and being heavier, while men were less likely to change their eating habits, report reduced appetite, eat a more monotonous diet, and be underweight [27].

Studies in community and population-based samples also suggest differential influence of OD on diet by age and across males and females, although large systematic investigations are still lacking. In community-based sample of free-living older women in a Northeast U.S. county, those with measured OD reported greater consumption of high fat/sweet foods than those without this dysfunction [28]. In a cohort study of 359 community-dwelling older adults in the Netherlands, self-reported OD provided more information than measured olfactory function to explain poorer appetite and diet quality [29]. In nationally representative samples from Korea and the US, the association between OD, diet and diet-related outcomes also showed sex and age effects independent of multiple demographic, health, and lifestyle variables [30,31]. Specifically, across nearly 25,000 Korean adults ages 25 to 90 years old, measured OD was associated with lower fat intake but greater carbohydrate intake in younger women and less protein intake in younger men [30]. In the nationally-representative NHANES sample of adults in the U.S., measured or self-rated OD was associated with lower BMI in older men (≥65 years) but higher BMI in younger women (40–64 years), again suggesting that differential dietary responses across age and across males and females might be at play [31].

In newer research, nutritional epidemiology has demonstrated the added value of examining the overall pattern of the diet, in addition to examining individual food groups or food components that constitute a healthy dietary pattern or a dietary quality index. Based on extensive evidence analyses, national and international dietary guidelines including those from the World Health Organization and the U.S. Dietary Guidelines have also increasingly focused on overall diet quality, with a higher quality diet being described in terms of a priori indices [32] or as being lower in energy density [33], and high in diversity [34] including diversity in types of vegetables from all subgroups (dark green; red and orange; beans, peas, and lentils; starchy; and other) [35]. While multiple studies have investigated the association of OD with energy intake and food groups, only a few have examined associations with diet quality, including a cross-sectional study of a convenience sample of adults from an Otolaryngology practice in Dresden, Germany [36], a cross-sectional analysis of free-living Dutch older adults [29], and a five-year longitudinal study of 550 U.S. older adults [37]. These studies consistently reported poor diet quality among those with poor olfactory function, but provided limited inference regarding age and sex-specific associations, as well as the underlying food components and characteristics driving the observed associations with diet quality.

Here, we examined associations between self-reported OD and diet quality in a nationally representative sample of US adults using data from questions included in the 2011–2014 NHANES. Self-reported olfactory function based on a single question, or multiple questions as in NHANES, has been shown to correspond reasonably well with measured function [12,16,17,18]. Additionally, subjective assessments of smell alterations are clinically relevant as they are the only means to capture individuals with smell distortions such as phantosmia (smell hallucinations) or parosmia (altered perceptual quality), intermittent smell losses, and those who likely experience an impact of smell alteration from a health or functional perspective.

The present detailed analyses complement a limited analysis of some of the US NHANES data reported elsewhere [38]. Here, our study team of experts in chemosensation, nutrition, statistics and epidemiology worked collaboratively to develop and test the hypothesis that self-reported OD would be associated with diet quality, and that the magnitude and direction of the associations would vary by age and sex. Specifically, we hypothesized that self-reported OD would be associated with higher energy density and poorer diet quality, including lower consumption of foods needed in an adequate diet (e.g., fruits, vegetables, and whole grains) and higher consumption of nutrients that require moderation (e.g., added sugars, saturated fats). Further, given the evidence summarized above, we hypothesized that younger women with OD would report the poorest diet quality whereas men would report a more monotonous diet, reflected as lower dietary diversity [26,27].

## 2. Materials and Methods

### 2.1. Data Source and Study Participants

The NHANES is a cross-sectional study conducted each year in the U.S. by the National Center for Health Statistics (NCHS) to examine the health and nutritional status of a nationally representative sample of noninstitutionalized U.S. civilian residents [39]. Data for the U.S. NHANES are collected from home interviews as well as physical examinations and interviews at mobile examination centers (MEC) [39]. The 2011–2014 NHANES administered chemosensory assessments to all eligible participants 40 years of age and older [40]. The chemosensory protocol consisted of a brief at-home chemosensory questionnaire (CSQ) as well as taste and smell examinations (CSX) completed in the MEC [41]. Dietary interviews for the U.S. NHANES 2011-2014 were collected as part of What We Eat in America (WWEIA) [42]. NHANES participants completed two 24 h dietary recalls; the first was collected in-person at the MEC, and the second was collected 3–10 days later by phone.

#### Analytic Sample

The current study utilized data from the CSQ and the first day dietary interview in the NHANES 2011–2014; data from the CSX will be reported elsewhere. The 2011–2014 NHANES administered the CSQ to all eligible participants 40 years of age and older (*n* = 7418) [40]. Survey response rate for the CSQ was 100% (*n* = 7418) but 20 participants did not answer the self-reported smell questions in the CSQ; 1031 participants had missing or incomplete dietary data. Participants who were pregnant or breastfeeding were excluded from analysis (*n* = 11), and thus our analytical sample was limited to non-pregnant, non-lactating participants aged 40 years and older who completed the self-reported smell questions in the CSQ and had a reliable first day 24 h dietary recall (N = 6356). The NHANES survey was approved by the National Center for Health Statistics (NCHS) Research Ethics Review Board, and all NHANES participants provided informed consent [43].

### 2.2. Olfactory Function

In the home interview, the CSQ asked questions regarding smell-related problems, treatments, and related health conditions [44]. As part of the process to include this assessment within NHANES, experts in chemosensation content-validated the questions, which were then tested to ensure consistency in participant understanding, processing and interpretation [40].

Here, we defined self-reported OD as acknowledgement of one or more of the following: a smell problem in the last 12 months, worse ability to smell relative to age 25, or smelling a phantom odor (including unpleasant, bad, or burning odor when nothing was there). Participants who did not report OD were considered as normosmic (having normal sense of smell). Previously, an index to classify OD based on these three questions has been shown to associate better with measured olfactory function [45] as well as other diagnosed conditions according to an evidence analysis [11]. From a conveniences sample of adults, the yes/no classification of OD showed excellent test–retest reliability over 6 months [46]. Self-rated OD associated with known risk factors of OD in previous analysis of the U.S. NHANES [47], with reasonable specificity (78.1%) and modest sensitivity (54.4%) in identifying anosmia/severe hyposmia [40]. This specificity/sensitivity pattern is expected of rarely measured conditions, such as OD [48].

### 2.3. Dietary Outcomes

First-day dietary interviews were conducted by trained dietary interviewers following the USDA Automated Multiple-Pass Method (AMPM) and using measuring guides to estimate portion sizes [49]. Participants recalled the amounts of foods and beverages consumed over the previous 24 h, providing detailed descriptions of each food/beverage item, such as when and where the item was consumed, any additions to the items, and any food/beverage combinations. The public domain nutrient intake data were computed from the USDA’s Food and Nutrient Database for Dietary Studies (FNDDS) versions 2011–2012 and 2013–2014, which were used to convert food and beverage items consumed in *WWEIA* into gram amounts and calculate energy and nutrient values [50]. The 2011–2012 and 2013–2014 Food Patterns Equivalents Databases (FPED) were used to determine food patterns equivalents [51].

Dietary outcomes examined included 24 h energy intake, energy density (ED) of foods, dietary diversity, percent energy from total fats, saturated fats, added sugars, and alcoholic beverages, and total, moderation and adequacy scores for HEI. Energy intake, total grams of food, and grams of total fats, saturated fats, and alcohol were taken from the 2011–2014 NHANES public domain data [52], and added sugars was taken from the FPED [53]. We calculated the ED of foods (kcal/gm), which included all foods and food combinations, and excluded beverages including alcohol and water [54]. Dietary diversity was defined as the number of unique food and beverage codes consumed, excluding plain water, in quantities of at least 15 g [55,56,57].

As an overall measure of diet quality, we computed the Healthy Eating Index 2015 (HEI–2015) for each study participant [58,59]. The HEI–2015 uses least-restrictive standards to set maximum scores for nine *adequacy* (total vegetables, greens and beans, total fruits, whole fruits, whole grains, dairy, total protein foods, seafood and plant proteins, and fatty acids) and four *moderation* (refined grains, sodium, saturated fats, and added sugars) components. All components, except fatty acids, saturated fats, and added sugars, are scored based on nutrient density per 1000 calories. A higher score of an adequacy component indicates higher intake, while a higher score of a moderation component indicates lower intake. The total HEI–2015 score (0 to 100) is the sum of all adequacy and moderation components, with a higher score indicating better diet quality. Analytic methods developed by the National Cancer Institute were used to compute the HEI–2015 scores [60].

### 2.4. Demographic, Clinical, and Lifestyle Characteristics

Demographic: Self-reported demographic variables included age (years), race/ethnicity (Mexican American, other Hispanic, non-Hispanic white, non-Hispanic black, non-Hispanic Asian, or other race, including multiracial), sex (male/female), marital status (married and living with partner or not married), education (high school or less or more than high school), and ratio of family income to poverty.

Clinical: Trained health technicians obtained various body measures in the MEC, including height, weight, and waist circumference. Body mass index (kg/m^2^) was calculated from measured height and weight. Self-reported health was categorized as excellent/very good/good or fair/poor. Similar to previous reports [61,62,63], a chronic disease score, ranging from 0 to 4, was calculated from the number of “yes” responses to medically diagnosed diabetes, cancer (excluding non-melanoma skin cancer), stroke, or heart attack.

Lifestyle: NHANES participants were administered the well-validated Global Physical Activity Questionnaire (GPAQ) to briefly assess their physical activity. Physical activity was defined as self-report of engaging in at least 10 minutes of vigorous or moderate-intensity work and/or recreational activity at least three days in a week [64]. History of heavy drinking was assessed as self-report of 4 (for females) or 5 (for males) alcoholic beverages every day for any period of time [65]. Smoking status was classified as never smokers (never smoked 100 cigarettes in lifetime), former smokers, or current smokers.

### 2.5. Statistical Analysis

Participant characteristics and dietary measures were summarized by olfactory function category (i.e., normosmic versus OD). Descriptive analyses of differences in participant characteristics and dietary measures between the two groups were tested using survey-weighted chi-square tests (for categorical variables) or *t*-tests (for continuous variables). Given known differences in OD and dietary intake by age and sex [26,29,30], we created age/sex categories (males 40–64 years, males ≥65 years, females 40–64 years, and females ≥65 years) a priori to stratify associations between olfactory function and dietary measures. Supplementary Table S1 summarizes the demographic, clinical, and lifestyle characteristics by the four age/sex categories. Multiple linear regression models, overall and by age/sex categories, were used to estimate associations between olfactory function and dietary measures, with results reported as adjusted mean difference estimates and 95% confidence intervals (CI) after adjusting for covariates [66]. We computed analyses using three adjusted models: the first controlled for age, sex, income, education level, race, and smoking status; the second controlled for all variables included in the first model, plus chronic disease score; and the third controlled for all variables included in the second model, plus physical activity. Supplementary Tables S2 and S3 provide the third model, which is also adjusted for BMI. All covariates included in the regression models were selected *a priori* based on the literature [47]. We tested for interaction between self-reported olfactory function and age/sex category by including an interaction term (self-reported olfactory function × age/sex category) in the final multivariable models. To examine effect modification by age and sex, we stratified the linear regression analyses by age/sex categories. We conducted additional analyses adjusting for the day of the recall (weekday or weekend) as well as the season (November–April vs. May–October).

In sensitivity analyses, we excluded over-reporters (*n* = 2193; 24 h energy intake/24 h basal energy expenditure >1.4) and under-reporters (*n* = 722; 24 h energy intake/24 h basal energy expenditure < 0.7) of energy intake [67,68], never drinkers (*n* = 2661; those who reported never consuming ≥12 alcoholic drinks in any one year or in their lifetime, or those who reported consuming no alcoholic beverages over the past 12 months), and participants who reported consuming no energy from alcoholic beverages in the first day 24 h dietary recall (*n* = 4939).

All statistical analyses were conducted using Statistical Analysis Software (SAS) version 9.4 (SAS Institute, Cary, NC, USA) and its survey data analyses procedures. All descriptive and multiple linear regression analyses were sample-weighted and accounted for the complex stratified, multistage, cluster sampling design of NHANES [69,70]. All *p*-values were two-sided and those < 0.05, without adjustment for multiple comparisons, were considered statistically significant.

## 3. Results

A total of 6356 participants ≥40 years of age were included in the analytic sample. OD was self-reported by 1399 participants and was associated with being older and having a lower ratio of family income to the poverty line (i.e., those with greater incomes were less likely to report dysfunction) (Table 1). Participants with self-reported OD were also less likely to be non-Hispanic Asian than participants who were normosmic.

Self-reported OD was associated with a higher BMI, greater waist circumference, and higher chronic disease score. Participants with OD were more likely to report their general health as fair/poor, compared to participants who were normosmic. Self-reported OD was associated with a greater likelihood of history of heavy drinking and a lower likelihood of being a never smoker.

**Figure 1:**
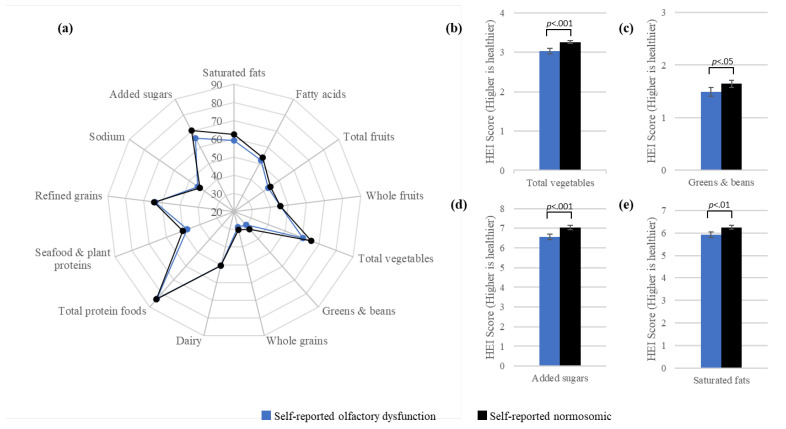
Visualization of the HEI–2015 component scores by olfactory function among NHANES 2011–2014 participants, aged 40 years and older. The radar graph (**a**) displays mean HEI–2015 component scores as a percentage of the maximum component score according to self-reported olfactory function, while the bar graphs (**b–e**) show significant differences in mean component scores by olfactory function for total vegetables (**b**), greens and beans (**c**), added sugars (**d**), and saturated fats (e). Lower scores correspond to lower intake for total vegetables (**b**) and greens and beans (**c**), whereas lower scores correspond to higher consumption for added sugars (**d**) and saturated fats (**e**).

Figure 1a displays the survey-weighted mean HEI–2015 component scores as a percentage of the maximum component score. Patterns of the mean component scores were similar for participants with and without self-reported OD. For both self-reported OD and normosmia, the lowest reported mean component scores were for greens and beans and whole grains, and the highest reported component scores were for total protein foods. For both groups, mean scores were 50% or less of the maximum component scores for total fruits, whole fruits, greens and beans, whole grains, seafood and plant proteins, and sodium. Figure 1b–e indicate that compared to normosmic participants, participants with OD had significantly lower scores or lower intake for total vegetables (b) and greens and beans (c), as well as lower scores corresponding to higher consumption for added sugars (d) and saturated fats (e).

In general, OD was associated with lower HEI–2015 (i.e., worse diet quality) as well as greater intake of foods with higher energy density, but patterns of significance varied across sex and age groups (Table 2). Middle-aged (40–64 years) and older (≥65 years) males with OD reported consuming diets highest in energy density; the lowest energy density diets were seen in normosmic older women. Likewise, women with OD reported greater percentage energy from total fat, compared to normosmic women. OD was associated with lower dietary diversity only among older men, whereas it was associated with lower HEI–2015 scores in all age groups except older women.

Lower HEI–2015 in adults reporting OD were related to different HEI components (moderation, adequacy) and related food groups, and also showed differences by sex and age groups (Table 2). Women ages 40–64 with OD had the lowest moderation scores, which were significantly lower when compared to similarly aged normosmic women. In the moderation grouping, the highest intake of energy from added sugars was reported by adults ages 40–64 (both women and men) with OD. In addition, in the moderation grouping, higher intake of energy from saturated fats was reported by women (both age groups) with OD, as compared to their normosmic counterparts. In the adequacy grouping, men (of both age groups) with OD had lower scores as compared to normosmic men. Some of this could have been driven by lower consumption of total fruits, which was reported by all adults with OD, except for men aged 40–64, who had the lowest intake of total fruits. Women 65 years and over with OD also reported lower intake of total and leafy green vegetables than did normosmic women of similar ages.

Energy intake from alcoholic beverages, which is not part of the HEI–2015, also differed by OD group. Men ages 40–64 reported the highest % energy from alcoholic beverages, and women 65 and over reported the lowest intake, irrespective of olfactory status, whereas men ages 65 and over and women ages 40–64 with OD reported lower intakes of energy from alcoholic beverages than their normosmic counterparts of similar age.

Across the entire sample, the survey-weighted multiple regression analyses (Table 3) generally supported the findings seen in Table 2. Self-reported OD was associated with intake of higher energy dense foods, poorer diet quality, and higher percent energy from total fat, even in fully adjusted models that controlled for demographic, health, and lifestyle factors. With respect to diet quality, OD was associated with lower HEI–2015 total scores, moderation scores, and adequacy scores in unadjusted models. In models adjusted for demographic, clinical, and lifestyle characteristics, the associations remained significant for HEI–2015 moderation scores and marginally significant for HEI–2015 total scores. Lower moderation scores with OD appeared to be driven by greater intakes of saturated fat and added sugars, which were significant even in the fully adjusted models. Neither the adequacy grouping nor its components differed by olfactory status in the fully adjusted models. Dietary diversity was not different across the entire group in the unadjusted or the adjusted models containing co-variates.

The survey-weighted multiple regression analyses stratified by age/sex category (Table 4) showed that the intake of more energy dense foods associated with OD was seen only in men and women ages 65 and over (*p* _interaction_ = 0.21). Most associations remained significant in women ages 40–64 with OD who also had lower HEI-2015 moderation scores and higher percent energy from saturated fat and total fat, as well as lower percent energy from alcoholic beverages than normosmic women ages 40–64 (*p*-values for interaction ranged from 0.07 to 0.31). Men ages 40–64 with OD had higher percent energy from added sugars than similarly aged normosmic men, while women ages 65 and over with OD reported lower intake of total vegetables than normosmic women in the same age group.

## 4. Discussion

This in-depth analysis of the 2011–2014 U.S. NHANES found that adults 40 years and older with self-reported olfactory dysfunction (OD) reported consuming foods with higher energy density and lower diet quality, with the latter appearing to be driven by greater consumption of saturated fats and added sugars as well as lower consumption of vegetables. Age- and sex-stratified analyses revealed that older adults (≥65 years) with OD drove most of the energy density findings while women ages 40–64 with OD drove most of the findings relating to energy intake from total and saturated fats. In similar controlled analyses, women ages 65 and over with OD reported lower intake of vegetables and men ages 40–64 reported greater intake of energy from added sugars. OD was associated with less dietary diversity only in men over 65, but the results failed to reach significance in models adjusted for covariates.

One of the dietary outcomes of interest in this study was the energy density of foods, which excluded energy density calculated from beverages including alcoholic drinks. Greater energy density from foods is largely a result of greater consumption of fat, which is the most energy-dense macronutrient. Analysis of a large cohort study in the UK show that diets with energy density are associated with greater intakes of fat, sugars, refined carbohydrates and lower intakes of fruits, vegetables, and whole grains [71], as the water content of fruits and vegetables substantially lowers their energy density. Similarly, newly published analyses of the U.S. NHANES data also show that more energy dense diets are associated with lower intakes of fruits and vegetables and higher intakes of added sugars and fats [72]. Our findings that adults with OD consumed more energy-dense diets are generally in line with currently observed and previously reported associations between OD and lower fruit and/or vegetable consumption [28]; however, they conflict with findings from the Korean NHANES data, where OD was associated with lower fat intake among young women and lower protein intake among young men [30]. Our findings also differ from experimental findings showing olfactory priming increases the selection of energy dense desserts [73], although appetitive effects of acute priming may represent a distinct mechanism from the influence of chronic dysfunction on habitual food choice. Our data may be explained by a tendency to compensate for chronic OD by seeking positive primary taste qualities (sweet, salty) and the positive texture components of fat, which could in turn explain the increased risk of weight gain [24,25,26] and CVD risk [31] among those with OD. Of note, we observed a difference in energy density ranging from 0.10 to 0.12 kcal/g (among older men/women with and without OD), which represents a difference of 140 to 168 kcal daily, if we assume adults consume approximately 1400 g of food per day.

Diets high in energy density are generally lower in diet quality [74,75], as has been previously observed with the U.S. NHANES dietary data [76]. In agreement, we observed that those with OD had diets that were more energy dense, in addition to being lower in diet quality. Our diet quality findings among U.S. adults 40 years and older largely conform with findings from findings from Dutch and Australian older adults, which also reported poorer diet quality related to OD [29,37]. Notably, analyses from the Korean NHANES [30] suggest that dietary associations with OD were particularly evident among younger and middle-aged women. Thus, our analysis of U.S. NHANES data continues to support age and sex differences in the effect of OD on diet quality in adults. Another dietary characteristic, which is associated with diet quality and commonly used as its proxy, is dietary diversity, especially diversity in vegetable and fruit consumption [35]. Eating a variety of foods was historically a component of the Dietary Guidelines up until 1995, and thus part of the scoring components of the earliest versions of Healthy Eating Index [77]. Consistent with previous reports [26,27], we found that men 65 and older with OD reported lower diet diversity; this finding did not retain statistical significance in multivariate models that adjusted for demographic, lifestyle, and health characteristics. However, it is worth noting that the dietary diversity measure used in our study was relatively crude, and thus a more nuanced operationalization of dietary diversity may present a different picture. Notably, we also failed to find an association between OD and total energy intake in either age group or in men and women.

Energy intake from alcoholic beverages was lower with OD among men ages 65 and older and women ages 40–64, but only remained significant among the women in the fully adjusted models. Alcoholic beverages present a complex perceptual mixture of odor-active volatiles and bitterness, sweetness, oral burn and drying that varies with the level of ethanol [78]. Greater olfactory input can enhance the enjoyment of ethanol [79], as may be intuitive to anyone who has sniffed their wine glass, or savored a peaty whisky. This suggests individuals with OD may obtain less sensory pleasure from alcoholic beverages, especially young women, who are known to report higher levels of burning and displeasure in general from ethanol even with self-reported normal sense of smell [80]. That said, the pharmacological reward from ethanol would be unaffected by OD. Thus, more work is needed to determine if there may be a shift in the type of alcoholic beverage consumed by those with OD who continue to drink alcohol (e.g., if neat vodka may replace aged whisky or red wine, if the olfactory aspects are absent).

Our finding that OD is related to selection of foods of higher energy density and lower in diet quality has important clinical and public health implications. While reducing dietary energy density can aid in weight loss [81], consuming a more energy-dense diet increases the risks of excessive adiposity [82], obesity-related cancers [83], cardiovascular disease and greater overall mortality [71]. Similarly, low diet quality, as reflected by lower HEI–2015 scores, has been associated with increased risk of several adverse health outcomes, including some types of lung cancer [84], depression [85], untreated dental caries [86], a lower level of frailty in older adults [87], and lower risk of all-cause mortality [88]. The public health burden related to OD and its downstream consequences on diet and health is very likely to magnify dramatically in the wake of COVID-19.

Loss of sense of smell is a hallmark sign of COVID-19 [1,2,3,4,5], emerging early in the disease course and affecting most infected individuals. For most COVID-19 patients, smell-related symptoms tend to resolve within a few weeks; however, 10–20% of individuals may experience chronic smell alterations, persisting beyond six months after onset [89,90,91,92,93]. Globally, the number of people who are living with chronic OD has suddenly increased by several million individuals and will continue to increase, owing to post-acute sequelae of COVID-19 (PASC). While our study findings shed important light on potential target areas and groups for nutritional intervention/counseling for OD, longitudinal studies among individuals living with PASC are needed to better address evidence gaps regarding the nutritional impact and management of OD arising from COVID-19.

This study has several strengths. The U.S. NHANES chemosensory and dietary protocols are well-validated [46,94] and are administered by rigorously trained NHANES technicians and dietitians, thereby ensuring high data standard and quality. Our findings are generalizable to U.S. adults 40 years and older, as the data were collected from a large nationally representative population-based survey. In addition, in the present analysis, we were able to account for several known confounding variables when examining associations between self-rated olfaction and diet quality. Still, there are a few limitations worth noting. Although well-validated, 24-h dietary recalls are dependent on memory and prone to recall bias [95]. Even though there is random day-to-day variation in diet, as measured by single 24-h dietary recalls, this variation, sometimes referred to as classical measurement error, will increase the variance of estimated dietary group means in the descriptive analyses and estimated multiple linear regression coefficients of dietary-dependent variables in adjusted analyses but will not bias these estimates [95,96]. Separately, self-reported olfactory function may not be a sensitive indicator of true dysfunction, particularly if the dysfunction is mild [45]. However, a self-report measure would likely capture microsmics (individuals with mild dysfunction) who perceive an impact of smell alteration from a health or functional perspective [22]; in addition, self-report is the current standard of care for diagnosing individuals with phantosmia or parosmia, as no validated psychophysical tests exist to capture distortions in smell quality (e.g., when fresh fruit smells similar to garbage, due to neuronal miswiring post-infection). Here, the cross-sectional and observational design means we were unable to infer the causality and direction of associations. Lastly, even though we adjusted for a range of potential confounders, we cannot rule out confounding from unknown or unmeasured variables.

## 5. Conclusions

In conclusion, our findings from this large, cross-sectional, nationally representative data set are generally consistent with and replicate prior evidence that disrupted olfactory function has meaningful dietary implications. Furthermore, we add to existing literature by showing that olfactory dysfunction (OD) may have a more negative impact on diet in middle-aged women compared to similarly aged men, or older men and women. Given the recent surge in the number of people with olfactory problems due to COVID-19, our findings have increased public health relevance and may also have important implications for nutritional risk stratification and management among those affected by COVID-19 related OD.

## Figures and Tables

**Table 1:** Demographic, clinical, and lifestyle characteristics among the 2011–2014 NHANES study participants, ages 40 years and older †.

Demographic, Clinical, and Lifestyle Characteristics	All Participants	Self-Reported Olfactory Dysfunction ^a^	Self-Reported Normosmic	*p*-Value *
	*n* = 6356	*n* = 1399	*n* = 4957	
Age (years)	57.9 ± 0.2	58.6 ± 0.4	57.7 ± 0.2	**0.005**
Age/sex categories				
Males 40–64 years	34.5	7.7 (0.5)	26.9 (0.9)	0.5
Males ≥65 years	12.9	3.2 (0.2)	9.7 (0.4)	
Females 40–64 years	37.1	8.0 (0.5)	29.1 (0.8)	
Females ≥65 years	15.5	3.4 (0.3)	12.2 (0.4)	
Race				
Mexican American	6.3	6.1 (1.5)	6.3 (1.3)	**0.003**
Other Hispanic	4.9	4.6 (1.1)	5.0 (0.9)	
Non-Hispanic White	71.0	73.4 (3.0)	70.3 (2.9)	
Non-Hispanic Black	10.7	9.8 (1.7)	11.0 (1.6)	
Non-Hispanic Asian	4.7	2.9 (0.7)	5.2 (0.7)	
Other Race—Including Multi-Racial	2.3	3.1 (0.7)	2.1 (0.3)	
Sex				
Male	47.4	48.8 (1.6)	47.0 (0.9)	0.3
Female	52.6	51.2 (1.6)	53.0 (0.9)	
Education level				
High school or less	38.4	40.6 (2.6)	37.8 (2.1)	0.1
More than high school	61.6	59.4 (2.6)	62.2 (2.1)	
Marital status ^b^				
Married or living with a partner	65.6	63.2 (1.9)	66.3 (1.2)	0.2
Not Married	34.4	36.8 (1.9)	33.7 (1.2)	
Ratio of family income to poverty	3.1 ± 0.1	2.9 ± 0.1	3.2 ± 0.1	**<0.001**
Body mass index (kg/m^2^)	29.3 ± 0.2	30.0 ± 0.3	29.2 ± 0.2	**<0.001**
Waist circumference (cm)	101.4 ± 0.4	103.3 ± 0.7	100.9 ± 0.4	**<0.001**
Chronic disease score ^c^	0.35 ± 0.01	0.43 ± 0.02	0.33 ± 0.01	**<0.001**
Self-reported general health condition				
Excellent, very good, good	79.5	72.0 (2.1)	81.7 (1.1)	**<0.001**
Fair, Poor	20.5	28.0 (2.1)	18.3 (1.1)	
Physical activity ^d^				
Yes	60.4	59.1 (2.2)	60.8 (1.1)	0.4
No	39.6	40.9 (2.2)	39.2 (1.1)	
History of heavy drinking ^e^				
Yes	7.2	10.8 (2.3)	6.2 (1.1)	**<0.001**
No	92.8	89.2 (2.3)	93.8 (1.1)	
Smoking history				
Never smokers	51.7	45.0 (2.2)	53.7 (1.4)	**0.001**
Former smokers	29.9	33.5 (1.9)	28.9 (1.2)	
Current smokers	18.4	21.5 (1.5)	17.5 (1.1)	

† Data are presented as % (standard error of percent) for categorical variables and mean ± standard error of mean for continuous variables. † All statistics weighted. Percentages may not reflect the expected value due to sampling weights and survey-weighted analyses. † The following variables had missing values: education level (*n* = 6352), marital status (*n* = 6352), ratio of family income to poverty (*n* = 5854), body mass index (*n* = 6274), waist circumference (*n* = 6082), self-reported general health condition (*n* = 6048), history of heavy drinking (*n* = 6015), and smoking history (*n* = 6352). * *p*-values for differences between olfactory dysfunction and normosmic participants were obtained by Chi-square or two-tailed *t*-tests. **Bolded** items have a significant difference between olfactory dysfunction and normal function. ^a^ Self-reported dysfunction is defined as an affirmative response to either: a smell problem in the last 12 months, worse ability to smell since age 25, or smelling phantom odors. ^b^ Marital status is dichotomized as married or living with a partner and not married (widowed, divorced, separated, never married). ^c^ Chronic disease score is based on reported diabetes, cancer, stroke and heart attack. ^d^ Physical activity is defined as self-report of doing at least 10 minutes of vigorous or moderate-intensity work and/or recreational activity at least 3 days in a week. ^e^ Heavy drinking history is defined as self-reported history of ever consuming 4–5 drinks every day.

**Table 2:** Mean dietary attributes from a 24 h recall by age, sex, and self-reported olfactory function status among NHANES 2011–2014 participants, ages 40 years and older.

Dietary Measures	All Participant (*n* = 6356)	Males 40–64 Years			Males ≥ 65 Years			Females 40–64 Years			Females ≥ 65 Years		
		Olfactory Dysfunction (*n* = 412)	Normosmic (*n* = 1602)		Olfactory Dysfunction (*n* = 250)	Normosmic (*n* = 799)		Olfactory Dysfunction (*n* = 507)	Normosmic (*n* = 1709)		Olfactory Dysfunction (*n* = 230)	Normosmic (*n* = 847)	
	Mean ± SEM	Mean ± SEM	Mean ± SEM	*p*-Value *	Mean ± SEM	Mean ± SEM	*p*-Value *	Mean ± SEM	Mean ± SEM	*p*-Value *	Mean ± SEM	Mean ± SEM	*p*-Value *
Energy intake (kcal)	2057 ± 19	2497 ± 72	2521 ± 42	0.7	2139 ± 60	2087 ± 40	0.4	1856 ± 40	1788 ± 21	0.06	1643 ± 53	1599 ± 34	0.4
Energy density of foods (kcal/g) ^a^	1.88 ± 0.02	2.04 ± 0.04	1.95 ± 0.02	**0.006**	1.93 ± 0.05	1.80 ± 0.03	**<0.001**	1.90 ± 0.04	1.87 ± 0.03	0.4	1.85 ± 0.05	1.71 ± 0.03	**0.003**
HEI-2015 total score ^b^	53.2 ± 0.5	50.1 ± 0.8	51.8 ± 0.7	**0.032**	53.1 ± 1.3	55.2 ± 0.9	**0.023**	51.1 ± 0.9	53.6 ± 0.7	**<0.001**	56.7 ± 1.2	56.3 ± 0.7	0.7
HEI-2015 moderation score ^c^	23.8 ± 0.2	23.5 ± 0.4	23.7 ± 0.3	0.7	23.8 ± 0.6	24.6 ± 0.4	0.07	22.3 ± 0.3	23.9 ± 0.3	**<0.001**	24.5 ± 0.5	24.5 ± 0.3	0.9
HEI-2015 adequacy score ^d^	29.4 ± 0.4	26.6 ± 0.6	28.1 ± 0.5	**0.006**	29.3 ± 0.9	30.6 ± 0.6	**0.040**	28.8 ± 0.7	29.7 ± 0.5	0.09	32.1 ± 0.8	31.8 ± 0.5	0.6
% Energy from total fat	33.9 ± 0.2	34.1 ± 0.6	33.7 ± 0.4	0.4	34.4 ± 0.6	34.3 ± 0.5	0.8	35.0 ± 0.5	33.5 ± 0.3	**<0.001**	35.2 ± 0.7	33.6 ± 0.5	**0.016**
% Energy from saturated fat	10.8 ± 0.1	10.9 ± 0.2	10.8 ± 0.1	0.6	11.3 ± 0.3	11.0 ± 0.2	0.3	11.3 ± 0.3	10.5 ± 0.1	**<0.001**	11.3 ± 0.4	10.7 ± 0.2	**0.049**
% Energy from added sugar	12.2 ± 0.3	14.1 ± 0.6	12.0 ± 0.4	**<0.001**	11.2 ± 0.7	10.4 ± 0.4	0.1	13.6 ± 0.6	12.5 ± 0.5	**0.037**	11.9 ± 0.5	11.2 ± 0.4	0.2
% Energy from alcoholic beverages	4.37 ± 0.23	5.73 ± 0.65	5.80 ± 0.48	0.9	2.92 ± 0.52	4.16 ± 0.40	**0.016**	3.21 ± 0.50	4.44 ± 0.43	**0.006**	1.92 ± 0.49	1.89 ± 0.24	0.9
Total fruits (cup eq/1000 kcal)	0.52 ± 0.02	0.42 ± 0.04	0.41 ± 0.03	0.7	0.46 ± 0.04	0.61 ± 0.04	**<0.001**	0.47 ± 0.04	0.54 ± 0.03	0.037	0.64 ± 0.05	0.75 ± 0.04	**0.042**
Whole fruits (cup eq/1000 kcal)	0.40 ± 0.02	0.32 ± 0.04	0.30 ± 0.02	0.4	0.33 ± 0.04	0.45 ± 0.04	**<0.001**	0.38 ± 0.03	0.43 ± 0.03	0.1	0.49 ± 0.05	0.58 ± 0.04	0.07
Total vegetables (cup eq/1000 kcal)	0.91 ± 0.02	0.78 ± 0.07	0.85 ± 0.02	0.2	0.84 ± 0.09	0.91 ± 0.04	0.2	0.92 ± 0.06	0.96 ± 0.03	0.3	0.87 ± 0.05	1.04 ± 0.04	**0.001**
Dark leafy greens and beans (cup eq/1000 kcal)	0.15 ± 0.01	0.13 ± 0.03	0.13 ± 0.01	0.9	0.13 ± 0.03	0.14 ± 0.02	0.5	0.17 ± 0.03	0.18 ± 0.02	0.4	0.12 ± 0.02	0.16 ± 0.01	**0.026**
Whole grains (oz eq/1000 kcal)	0.53 ± 0.02	0.39 ± 0.04	0.46 ± 0.03	0.08	0.61 ± 0.07	0.63 ± 0.04	0.7	0.49 ± 0.04	0.50 ± 0.02	0.7	0.69 ± 0.07	0.70 ± 0.04	0.9
Dietary diversity ^e^	11.7 ± 0.1	11.6 ± 0.3	11.8 ± 0.2	0.4	11.9 ± 0.4	12.7 ± 0.2	**0.004**	11.3 ± 0.2	11.5 ± 0.2	0.3	11.9 ± 0.4	11.6 ± 0.2	0.4

Abbreviations: Healthy Eating Index 2015 score (HEI). * *p*-values for differences between dysfunction and normosmic participants were obtained by survey-weighted two-tailed *t*-tests. **Bolded** items have a significant difference between olfactory dysfunction and normal function. ^a^ *n* = 6349 (seven reported consuming only beverages). ^b^ Total score from 0–100; higher score indicates better diet quality. ^c^ Moderation score from 0–40; higher score indicates lower intake of moderation components. ^d^ Adequacy score from 0–60; higher score indicates higher intake of adequacy components. ^e^ Dietary diversity defined as the number of unique food and beverage codes reported in a 24 h recall, excluding plain water, in quantities of at least 15 g.

**Table 3:** Associations between self-reported olfactory function and dietary measures among NHANES 2011-2014 participants, ages 40 years and older, tested in survey-weighted regression models.

Dietary Measures	Unadjusted	Model 1 ^a^	Model 2 ^b^	Model 3 ^c^
	Mean Difference (95% CI)	Mean Difference (95% CI)	Mean Difference (95% CI)	Mean Difference (95% CI)
Energy intake	36.6 (−42.7, 115.9)	24.6 (−57.9, 107.1)	32.7 (−49.5, 115.0)	33.0 (−50.0, 115.6)
Energy density of foods (kcal/g)	0.08 (0.03, 0.13) **	0.06 (0.002, 0.12) *	0.06 (0.002, 0.12) *	0.06 (0.004, 0.11) *
HEI–2015 total score	−1.67 (−2.74, −0.61) **	−1.15 (−2.29, −0.01) *	−1.09 (−2.22, 0.05)	−1.07 (−2.19, 0.05)
HEI–2015 moderation score	−0.74 (−1.26, −0.22) **	−0.71 (−1.27, −0.16) *	−0.67 (−1.22, −0.12) *	−0.67 (−1.22, −0.11) *
HEI–2015 adequacy score	−0.94 (−1.72, −0.15) *	−0.43 (−1.21, 0.35)	−0.42 (−1.20, 0.36)	−0.41 (−1.18, 0.36)
% Energy from total fat	0.97 (0.30, 1.63) **	1.01 (0.25, 1.76) **	0.96 (0.21, 1.72) *	0.96 (0.22, 1.70) *
% Energy from saturated fat	0.48 (0.15, 0.80) **	0.48 (0.13, 0.83) **	0.47 (0.12, 0.82) **	0.47 (0.12, 0.81) **
% Energy from added sugar	1.30 (0.60, 2.00) ***	0.95 (0.29, 1.62) **	1.00 (0.33, 1.67) **	1.00 (0.33, 1.66) **
% Energy from alcoholic beverages	−0.64 (−1.47, 0.20)	−0.86 (−1.74, 0.03)	−0.77 (−1.66, 0.12)	−0.77 (−1.65, 0.12)
Total fruits (cup eq/1000 kcal)	−0.06 (−0.11, −0.01) *	−0.03 (−0.08, 0.02)	−0.03 (−0.08, 0.02)	−0.03 (−0.08, 0.02)
Whole fruits (cup eq/1000 kcal)	−0.04 (−0.08, 0.003)	−0.02 (−0.06, 0.03)	−0.02 (−0.06, 0.03)	−0.02 (−0.06, 0.03)
Total vegetables (cup eq/1000 kcal)	−0.08 (−0.16, 0.004)	−0.05 (−0.14, 0.03)	−0.06 (−0.14, 0.03)	−0.06 (−0.14, 0.03)
Dark leafy greens and beans (cup eq/1000 kcal)	−0.01 (−0.04, 0.01)	−0.004 (−0.03, 0.02)	−0.002 (−0.03, 0.03)	−0.002 (−0.03, 0.03)
Whole grains (oz eq/1000 kcal)	−0.03 (−0.08, 0.02)	−0.03 (−0.08, 0.02)	−0.03 (−0.09, 0.02)	−0.03 (−0.09, 0.02)
Dietary diversity Score ^d^	−0.19 (−0.50, 0.12)	−0.04 (−0.34, 0.26)	−0.02 (−0.32, 0.28)	−0.02 (−0.31, 0.28)

Abbreviations: Healthy Eating Index 2015 score (HEI). **Bolded** items indicate significant mean differences between olfactory dysfunction and normal function (* *p* < 0.05; ** *p* < 0.01; *** *p* < 0.001). ^a^ Adjusted for age, sex, income, education level, race/Hispanic origin, and smoking status. ^b^ Adjusted for all variables in Model 1 and chronic disease score. ^c^ Adjusted for all variables in Model 2 and physical activity. ^d^ Dietary diversity defined as the number of unique food and beverage codes reported in a 24 h recall, excluding plain water, in quantities of at least 15 g.

**Table 4:** Associations between self-reported olfactory function and dietary attributes by age and sex among NHANES 2011-2014 participants, ages 40 years and older, tested in survey-weighted regression models.

Dietary Measures	Males 40–64 Years	Males ≥65 Years	Females 40–64 Years	Females ≥65 Years
	Mean Difference ^a^ (95% CI)	Mean Difference ^a^ (95% CI)	Mean Difference ^a^ (95% CI)	Mean Difference ^a^ (95% CI)
Energy intake	−48.4 (−214.2, 117.4)	77.1 (−52.1, 206.3)	87.4 (−27.5, 202.4)	55.7 (−67.0, 178.3)
Energy density of foods (kcal/g)	0.06 (−0.04, 0.17)	0.10 (0.01, 0.19) *	0.01 (−0.08, 0.09)	0.12 (0.01, 0.23) *
HEI–2015 total score	−1.01 (−3.00, 0.99)	−1.34 (−4.35, 1.68)	−1.58 (−3.58, 0.41)	0.94 (−1.23, 3.10)
HEI–2015 moderation score	−0.02 (−1.11, 1.07)	−0.56 (−2.10, 0.98)	−1.62 (−2.47, −0.77) ***	0.12 (−1.01, 1.24)
HEI–2015 adequacy score	−0.99 (−2.21, 0.23)	−0.78 (−2.61, 1.06)	0.03 (−1.43, 1.50)	0.82 (−0.57, 2.21)
% Energy from total fat	0.17 (−1.24, 1.58)	0.02 (−1.41, 1.44)	1.90 (0.71, 3.10) **	1.41 (−0.35, 3.18)
% Energy from saturated fat	**0.02 (−0.65, 0.68)**	0.22 (−0.47, 0.92)	0.96 (0.31, 1.61) **	0.49 (−0.39, 1.37)
% Energy from added sugar	1.57 (0.49, 2.65) **	0.66 (−0.98, 2.31)	0.63 (−0.69, 1.95)	0.87 (−0.19, 1.93)
% Energy from alcoholic beverages	−0.02 (−1.76, 1.73)	−1.03 (−2.45, 0.39)	−1.71 (−3.18, −0.25) *	0.05 (−1.05, 1.15)
Total fruits (cup eq/1000 kcal)	0.03 (−0.08, 0.14)	−0.11 (−0.22, 0.00003)	−0.04 (−0.13, 0.05)	−0.09 (−0.23, 0.05)
Whole fruits (cup eq/1000 kcal)	0.04 (−0.05, 0.13)	−0.09 (−0.20, 0.01)	−0.02 (−0.10, 0.06)	−0.07 (−0.20, 0.07)
Total vegetables (cup eq/1000 kcal)	−0.06 (−0.19, 0.07)	−0.04 (−0.24, 0.17)	−0.01 (−0.15, 0.13)	−0.17 (−0.28, −0.05) **
Dark leafy greens and beans (cup eq/1000 kcal)	0.01 (−0.05, 0.08)	−0.01 (−0.07, 0.05)	0.01 (−0.05, 0.06)	−0.03 (−0.08, 0.02)
Whole grains (oz eq/1000 kcal)	−0.06 (−0.14, 0.03)	−0.05 (−0.19, 0.10)	−0.01 (−0.10, 0.08)	−0.004 (−0.14, 0.13)
Dietary diversity ^b^	−0.23 (−0.80, 0.34)	−0.38 (−1.17, 0.40)	0.16 (−0.27, 0.58)	0.37 (−0.53, 1.27)

Abbreviations: Healthy Eating Index 2015 score (HEI). **Bolded** items indicate significant mean differences between olfactory dysfunction and normal function (* *p* < 0.05; ** *p* < 0.01; *** *p* < 0.001). ^a^ Adjusted for age, income, education level, race/Hispanic origin, smoking status, chronic disease score, and physical activity. ^b^ Dietary diversity defined as the number of unique food and beverage codes reported in a 24 h recall, excluding plain water, in quantities of at least 15 g.

## Data Availability

The data are available to the public (https://www.cdc.gov/nchs/data_access/ftp_data.htm accessed on 13 October 2021).

## Supplementary Materials

The following are available online at https://www.mdpi.com/article/10.3390/nu13124561/s1, Table S1: Demographic, clinical, and lifestyle characteristics among the 2011–2014 NHANES study participants, ages 40 years and older, Table S2: Associations between self-reported olfactory function and dietary measures among NHANES 2011–2014 participants, ages 40 years and older, tested in survey-weighted regression models, Table S3: Associations between self-reported olfactory function and dietary attributes by age and sex among NHANES 2011–2014 participants, ages 40 years and older, tested in survey-weighted regression models.
