# Supplementary material for: Self-Reported Olfactory Dysfunction and Diet Quality: Findings from the 2011–2014 National Health and Nutrition Examination Survey (NHANES)
Source: Nutrients. 2021 Dec 20;13(12):4561. doi: 10.3390/nu13124561 (PMC8704378; doi:10.3390/nu13124561)
Supplement: Supplementary file 1 [file nutrients-13-04561-s001.zip › nutrients-1441209-supplementary.pdf]

Supplementary Table S1. Demographic, clinical, and lifestyle characteristics among the 2011-2014 NHANES study participants, ages 40 years and older†

Demographic, Clinical, and Lifestyle Characteristics	Males 40-64 years	Males ≥65 years	Females 40-64 years	Females ≥65 years	p-value*
	n=2014	n=1049	n=2216	n=1077	
Race					
Mexican American	2.7 (0.6)	0.5 (0.1)	2.6 (0.6)	0.5 (0.1)	**<0.0001**
Other Hispanic	1.8 (0.4)	0.5 (0.1)	2.1 (0.4)	0.7 (0.2)	
Non-Hispanic White	23.7 (1.3)	10.1 (0.5)	25.1 (1.4)	12.1 (0.5)	
Non-Hispanic Black	3.7 (0.6)	1.1 (0.2)	4.7 (0.7)	1.3 (0.2)	
Non-Hispanic Asian	1.7 (0.2)	0.5 (0.1)	1.9 (0.3)	0.7 (0.2)	
Other Race – Including Multi-Racial	0.9 (0.2)	0.3 (0.1)	0.8 (0.2)	0.2 (0.1)	
Education level					
High school or less	13.9 (1.0)	5.0 (0.3)	12.4 (0.8)	7.2 (0.4)	**<0.0001**
More than high school	20.7 (0.9)	7.8 (0.4)	24.7 (1.4)	8.3 (0.4	
Marital status[b]					
Married or living with a partner	24.4 (0.7)	9.9 (0.4)	23.7 (0.8)	7.7 (0.35)	
Not Married	10.1 (0.8)	3.0 (0.3)	13.4 (0.7)	7.9 (0.4)	
Ratio of family income to poverty	3.1 ± 0.1	3.2 ± 0.1	3.2 ± 0.2	2.7 ± 0.1	**<0.0001**
Body mass index (kg/m^2)	29.3 ± 0.2	28.5 ± 0.3	29.9 ± 0.3	28.8 ± 0.2	**<0.0001**
Waist circumference (cm)	104.2 ± 0.5	105.7 ± 0.7	98.6 ± 0.6	98.5 ± 0.7	**<0.0001**
Chronic disease score[c]	0.23 ± 0.01	0.70 ± 0.04	0.25 ± 0.01	0.57 ± 0.03	**<0.0001**
Self-reported general health condition					
Excellent, very good, good	28.1 (0.8)	10.1 (0.5)	28.8 (1.1)	12.5 (0.5)	0.1
Fair, Poor	6.3 (0.5)	3.0 (0.2)	7.7 (0.6)	3.5 (0.3)	
Physical activity[d]					
Yes	23.7 (0.9)	7.0 (0.4)	22.4 (0.8)	7.2 (0.4)	**<0.0001**
No	10.8 (0.6)	5.9 (0.3)	14.6 (0.6)	8.3 (0.4)	
History of heavy drinking[e]					
Yes	8.7 (0.7)	2.7 (0.3)	3.5 (0.4)	0.7 (0.1)	**<0.0001**
No	25.7 (0.9)	10.4 (0.5)	33.0 (0.9)	15.2 (0.5)	
Smoking history					
Never smokers	16.5 (0.8)	4.8 (0.4)	21.2 (1.0)	9.2 (0.4)	**<0.0001**
Former Smokers	9.7 (0.6)	6.8 (0.3)	8.1 (0.8)	5.2 (0.3)	
Current Smokers	8.3 (0.5)	1.3 (0.2)	7.7 (0.7)	1.1 (0.2)	

†Data are presented as % (standard error of percent) for categorical variables and mean ± standard error of mean for continuous variables.

†All statistics weighted. Percentages may not reflect the expected value due to sampling weights and survey-weighted analyses.

†The following variables had missing values: education level (N=6352), marital status (N=6352), ratio of family income to poverty (N=5854), body mass index (N=6274), waist circumference (N=6082), self-reported general health condition (N=6048), history of heavy drinking (N=6015), and smoking history (N=6352)

*p-values for differences between the four age/sex categories were obtained by Chi-square or two-tailed t-tests. Bolded items have significant difference across the four age/sex categories.

[b]Marital status is dichotomized as married or living with a partner–and not married (widowed, divorced, separated, never married).
[c]Chronic disease score is based on reported diabetes, cancer, stroke and heart attack
[d]Physical activity is defined as self-report of doing at least 10 minutes of vigorous or moderate-intensity work and/or recreational activity at least 3 days in a week.
[e]Heavy drinking history is defined as self-reported history of ever consuming 4-5 drinks every day.

Supplementary Table S2. Associations between self-reported olfactory function and dietary measures among NHANES 2011-2014 participants, ages 40 years and older, tested in survey-weighted regression models

Dietary Measures	Unadjusted Mean difference (95% CI)	Model 1[a] Mean difference (95% CI)	Model 2[b] Mean difference (95% CI)	Model 3[c] Mean difference (95% CI)	Model 4[d] Mean difference (95% CI)
Energy intake	36.6 (−42.7, 115.9)	24.6 (−57.9, 107.1)	32.7 (−49.5, 115.0)	33.0 (−50.0, 115.6)	33.9 (−49.9, 117.6)
Energy density of foods (kcal/g)	**0.08 (0.03, 0.13)****	**0.06 (0.002, 0.12)***	**0.06 (0.002, 0.12)***	**0.06 (0.004, 0.11)***	**0.06 (0.00, 0.11)***
HEI-2015 total score	**−1.67 (−2.74, −0.61)****	**−1.15 (−2.29, −0.01)***	−1.09 (−2.22, 0.05)	−1.07 (−2.19, 0.05)	−0.84(−1.93, 0.26)
HEI-2015 moderation score	**−0.74 (−1.26, −0.22)****	**−0.71 (−1.27, −0.16)***	**−0.67 (−1.22, −0.12)***	**−0.67 (−1.22, −0.11)***	**−0.56 (−1.11, −0.01)***
HEI-2015 adequacy score	**−0.94 (−1.72, −0.15)***	−0.43 (−1.21, 0.35)	−0.42 (−1.20, 0.36)	−0.41 (−1.18, 0.36)	−0.28 (−1.04, 0.49)
% Energy from total fat	**0.97 (0.30, 1.63)****	**1.01 (0.25, 1.76)****	**0.96 (0.21, 1.72)***	**0.96 (0.22, 1.70)***	**0.90 (0.16, 1.65)***
% Energy from saturated fat	**0.48 (0.15, 0.80)****	**0.48 (0.13, 0.83)****	**0.47 (0.12, 0.82)****	**0.47 (0.12, 0.81)****	**0.44 (0.09, 0.78)****
% Energy from added sugar	**1.30 (0.60, 2.00)*****	**0.95 (0.29, 1.62)****	**1.00 (0.33, 1.67)****	**1.00 (0.33, 1.66)****	**1.03 (0.36, 1.71)***
% Energy from alcoholic beverages	−0.64 (−1.47, 0.20)	−0.86 (−1.74, 0.03)	−0.77 (−1.66, 0.12)	−0.77 (−1.65, 0.12)	−0.69 (−1.59, 0.22)
Total fruits (cup eq/1000 kcal)	**−0.06 (−0.11, −0.01)***	−0.03 (−0.08, 0.02)	−0.03 (−0.08, 0.02)	−0.03 (−0.08, 0.02)	−0.02 (−0.07, 0.03)
Whole fruits (cup eq/1000 kcal)	−0.04 (−0.08, 0.003)	−0.02 (−0.06, 0.03)	−0.02 (−0.06, 0.03)	−0.02 (−0.06, 0.03)	−0.01 (−0.06, 0.03)
Total vegetables (cup eq/1000 kcal)	−0.08 (−0.16, 0.004)	−0.05 (−0.14, 0.03)	−0.06 (−0.14, 0.03)	−0.06 (−0.14, 0.03)	−0.05 (−0.14, 0.03)
Dark leafy greens and beans (cup eq/1000 kcal)	−0.01 (−0.04, 0.01)	−0.004 (−0.03, 0.02)	−0.002 (−0.03, 0.03)	−0.002 (−0.03, 0.03)	0.00 (−0.03, 0.03)
Whole grains (oz eq/1000 kcal)	−0.03 (−0.08, 0.02)	−0.03 (−0.08, 0.02)	−0.03 (−0.09, 0.02)	−0.03 (−0.09, 0.02)	−0.02 (−0.08, 0.03)
Dietary diversity Score[e]	−0.19 (−0.50, 0.12)	−0.04 (−0.34, 0.26)	−0.02 (−0.32, 0.28)	−0.02 (−0.31, 0.28)	0.02 (−0.27, 0.32)

Abbreviations: Healthy Eating Index 2015 score (HEI).

Bolded items indicate significant mean differences between olfactory dysfunction and normal function (*p<0.05; **p<0.01; ***p<0.001).

[a]Adjusted for age, sex, income, education level, race/Hispanic origin, and smoking status.

[b]Adjusted for all variables in Model 1 and chronic disease score.

[c]Adjusted for all variables in Model 2 and physical activity.

[d]Adjusted for all variables in Model 3 and BMI.

[e] Dietary diversity defined as the number of unique food and beverage codes reported in a 24 hour recall, excluding plain water, in quantities of at least 15 g.

Supplementary Table S3. Associations between self-reported olfactory function and dietary attributes by age and sex among NHANES 2011-2014 participants, ages 40 years and older, tested in survey-weighted regression models

Dietary Measures	Males 40-64 years	Males ≥65 years	Females 40-64 years	Females ≥65 years
	**Mean difference[a] (95% CI)**	**Mean difference[a] (95% CI)**	**Mean difference[a] (95% CI)**	**Mean difference[a] (95% CI)**
Energy intake	−45.9 (−211.6, 119.9)	50.84 (−81.4, 183.0)	77.05 (−36.8, 190.9)	56.8 (−70.4, 184.0)
Energy density of foods (kcal/g)	0.06 (−0.04, 0.17)	0.09 (0.00, 0.18)	−0.01 (−0.09, 0.08)	**0.13 (0.02, 0.24)***
HEI-2015 total score	−0.90 (−2.81, 1.01)	−1.46 (−4.38, 1.46)	−0.84 (−2.76, 1.08)	0.71 (−1.45, 2.88)
HEI-2015 moderation score	0.02 (−1.03, 1.07)	−0.67 (−2.17, 0.83)	**−1.31 (−2.13, −0.48)****	0.05 (−1.06, 1.15)
HEI-2015 adequacy score	−0.92 (−2.11, 0.27)	−0.80 (−2.57, 0.98)	0.47 (−0.97, 1.90)	0.67 (−0.72, 2.06)
% Energy from total fat	0.17 (−1.21, 1.55)	0.06 (−1.41, 1.53)	**1.73 (0.48, 2.99)****	1.39 (−0.36, 3.14)
% Energy from saturated fat	0.02 (−0.64, 0.68)	0.31 (−0.38, 1.00)	**0.85 (0.21, 1.50)***	0.45 (−0.44, 1.33)
% Energy from added sugar	**1.61 (0.52, 2.69)****	0.42 (−1.23, 2.07)	0.57 (−0.80, 1.93)	1.00 (−0.08, 2.09)
% Energy from alcoholic beverages	0.06 (−1.68, 1.81)	−1.06 (−2.50, 0.38)	**−1.83 (−3.00, −0.06)***	0.03 (−1.10, 1.16)
Total fruits (cup eq/1000 kcal)	0.04 (−0.07, 0.15)	−0.10 (−0.21, 0.01)	−0.01 (−0.11, 0.08)	−0.09 (−0.24, 0.06)
Whole fruits (cup eq/1000 kcal)	0.04 (−0.05, 0.13)	−0.09 (−0.20, 0.02)	0.00 (−0.08, 0.08)	−0.07 (−0.21, 0.07)
Total vegetables (cup eq/1000 kcal)	−0.06 (−0.19, 0.07)	−0.03 (−0.24, 0.18)	0.01 (−0.13, 0.15)	**−0.17 (−0.28, −0.05)****
Dark leafy greens and beans (cup eq/1000 kcal)	0.01 (−0.05, 0.08)	−0.01 (−0.07, 0.05)	0.01 (−0.04, 0.07)	−0.03 (−0.08, 0.02)
Whole grains (oz eq/1000 kcal)	−0.05 (−0.14, 0.04)	−0.03 (−0.18, 0.11)	0.01 (−0.08, 0.10)	−0.01 (−0.15, 0.13)
Dietary diversity[b]	−0.22 (−0.80, 0.36)	−0.43 (−1.23, 0.38)	0.23 (−0.19, 0.65)	0.32 (−0.60, 1.25)

Abbreviations: Healthy Eating Index 2015 score (HEI).

Bolded items indicate significant mean differences between olfactory dysfunction and normal function (*p<0.05; **p<0.01; ***p<0.001).

[a]Adjusted for age, income, education level, race/Hispanic origin, smoking status, chronic disease score, physical activity, and BMI.

[b] Dietary diversity defined as the number of unique food and beverage codes reported in a 24 hour recall, excluding plain water, in quantities of at least 15 g.
